# Reduced entomopathogen abundance in *Myrmica* ant nests—testing a possible immunological benefit of myrmecophily using *Galleria mellonella* as a model

**DOI:** 10.1098/rsos.150474

**Published:** 2015-10-21

**Authors:** Sämi Schär, Louise L. M. Larsen, Nicolai V. Meyling, David R. Nash

**Affiliations:** 1 Centre for Social Evolution, Department of Biology, University of Copenhagen, Universitetsparken 15, 2100 Copenhagen Ø, Denmark; 2 Department of Plant and Environmental Sciences, University of Copenhagen, Thorvaldsensvej 40, 1871 Frederiksberg C, Denmark

**Keywords:** symbiosis, immunocompetence, hygiene

## Abstract

Social insects such as ants have evolved collective rather than individual immune defence strategies against diseases and parasites at the level of their societies (colonies), known as social immunity. Ants frequently host other arthropods, so-called myrmecophiles, in their nests. Here, we tested the hypothesis that myrmecophily may partly arise from selection for exploiting the ants’ social immunity. We used larvae of the wax moth *Galleria mellonella* as ‘model myrmecophiles’ (baits) to test this hypothesis. We found significantly reduced abundance of entomopathogens in ant nests compared with the surrounding environment. Specific entomopathogen groups (*Isaria fumosorosea* and nematodes) were also found to be significantly less abundant inside than outside ant nests, whereas one entomopathogen (*Beauveria brongniartii*) was significantly more abundant inside nests. We therefore hypothesize that immunological benefits of entering ant nests may provide us a new explanation of why natural selection acts in favour of such a life-history strategy.

## 1. Introduction

Group life has many advantages compared with a solitary lifestyle, and some of the ecologically most dominant organisms live in groups [1]. However, social life often comes at the cost of increased risk of infectious disease, as the frequent interactions between social organisms and the high densities in which they normally occur facilitate transmission of pathogens and parasites [2–4]. Some group-living animals have therefore evolved collective immune defence against diseases, known as social immunity [5]. Social insects have developed behavioural and chemical countermeasures against diseases in order to avoid infection and transmission [5]. A widespread behavioural strategy against diseases is hygienic behaviour such as allogrooming [6] and removal of dead corpses from the nest [7].

Ants frequently host other arthropod species that have evolved to live closely with them or even inside ants’ nests (inquilines) [8]. Little is known as to how inquilinism in distantly related invertebrates evolves. In myrmecophilous butterflies that do not live in ant nests as inquilines, pupation can still take place inside ant nests [9]. This could explain how inquilines arise, when selection favours the penetration of ant nests. Once inquilinism is established, benefits that the host ants provide to these myrmecophiles seem obvious: shelter, protection from natural enemies and often food. In consequence, like traditional parasites, inquilines may lose vital functions of their free-living ancestors, because selection for their maintenance is lost. For example, inquilines could lose their immunocompetence as they free-ride on the social immunity provided by the ants.

To examine the basis of this hypothesis, we studied the abundance of entomopathogens in nests of *Myrmica rubra* (Linnaeus) and *Myrmica ruginodis* (Nylander), both host ants of various myrmecophiles [10], using larvae of the wax moth *Galleria mellonella* (Linnaeus) as ‘model myrmecophiles’ (baits).

## 2. Material and methods

### 2.1 Collection of soil samples and soil baiting

A total of 166 soil samples from 13 sites (55 samples from *M. rubra* nests, 28 samples from *M. ruginodis* nests and 83 matched controls) were collected in August 2012 from sites in the area of northeastern Zealand (Denmark; table 1). Each sample was collected as a cylindrical core of approximately 10 cm depth and 5 cm diameter from the uppermost soil layer (including the soil surface) using a bulb planter (model: Gardena 3412). The bulb planter was rinsed in 70% ethanol and air-dried between samples. Samples were taken from nest cores of *M. rubra* group ants (*M. rubra* or *M. ruginodis*) as well as from a control point in the surrounding of the nest, together forming a sample pair. The ants were identified in the field by use of a 10× magnification hand lens. The control point was always chosen within a radius of 2 m from the ant nest, from the ant free point with the visually most similar type of soil compared with that of the ant nest. The soil was collected in plastic freezer bags and transferred to a 5°C climate chamber on the day of collection, where it was stored until analysis. All samples were processed by removing roots, stones and large pieces of wood, and homogenized by crushing soil clumps manually, and thoroughly mixing the sample, in plastic bags. Ants were removed using soft forceps sterilized in 70% ethanol, which were subsequently rinsed with distilled water between samples. All ants were kept in 96% ethanol as voucher specimens. Soil baiting was carried out as suggested in Meyling [11]. Moist soil from each individual sample point was distributed in even volumes into two plastic cups per sample, and each cup received 10 second- or third-instar (*ca* 10–15 mm) larvae of *G. mellonella*. The larvae came from a continuous culture kept at the University of Copenhagen and were heat-treated as described in Meyling & Eilenberg [12] prior to baiting in order to destroy the silk glands, so increasing their exposure to entomopathogens present in the soil by reducing the ability of the larvae to encapsulate inside webs. Soil samples were checked weekly during four weeks, dead larvae were rinsed with distilled water twice and isolated individually into 30 ml medicine cups containing a *ca* 2×2 cm piece of moist filter paper to maintain high humidity. Fungi emerging from dead *G. mellonella* larvae were classified to morphospecies level based on spore morphology using an Olympus BH-2 microscope at 100–400× magnification according to the key of Humber [13]. Spores from each detected morphospecies of entomopathogenic fungus per soil sample were transferred to standard selective media plates [11] until a clean culture could be established. Nematodes were identified as entomopathogenic when occurring in very large numbers, approximately replacing the biomass of the dead *G. mellonella* larva or when they showed ‘ambush behaviour’ [14]. If isolated nematodes were observed on cadavers, they were classified as ‘soil nematodes’ and not included. Nematodes were stored in 96% ethanol.

**Table 1.** Distribution and diversity of entomopathogens in *G. mellonella* baited soil collected from *Myrmica* ant nests and control samples per locality.

site	latitude (°N)	longitude (°E)	altitude (m)	no. samples (ant nest/control)	ant species	pathogens in soil from *Myrmica* ant nests	pathogens in surrounding soils (controls)
Vaserne	55.82	12.45	23	10/10	*M. rubra*	*M. brunneum*	*M. brunneum*
						*I. fumosorosea*	nematodes
Allerød Sø	55.87	12.36	45	9/9	*M. rubra*	*M. brunneum*	*M. brunneum*
						*B. bassiana*	*B. bassiana*
						*B. brongniartii*	*I. fumosorosea*
						*I. fumosorosea*	nematodes
Orehøjvej	55.80	12.32	39	1/1	*M. ruginodis*	none	none
Nymøllevej	55.82	12.30	28	10/10	*M. rubra*	*M. brunneum*	*M. brunneum*
							*I. fumosorosea*
							nematodes
Gadevang	55.97	12.27	41	5/5	*M. rubra*	none	*M. brunneum*
							nematodes
Klampenborgvej	55.77	12.56	11	4/4	*M. rubra*	*B. bassiana*	*M. brunneum*
						nematodes	*B. bassiana*
							*I. fumosorosea*
Dyrehavn	55.78	12.58	16	5/5	*M. rubra*	*M. brunneum*	*M. brunneum*
						nematodes	
Dyrehavn	55.78	12.58	15	2/2	*M. ruginodis*	*M. brunneum*	*M. brunneum*
						*I. fumosorosea*	*I. fumosorosea*
						nematodes	nematodes
Birkerød	55.83	12.45	37	2/2	*M. ruginodis*	*M. brunneum*	*M. brunneum*
						*B. brongniartii*	*B. bassiana*
							nematodes
Brødeskov	55.89	12.32	39	7/7	*M. ruginodis*	*M. brunneum*	*M. brunneum*
						*B. bassiana*	*I. fumosorosea*
						nematodes	nematodes
Allerød forest	55.87	12.37	54	9/9	*M. ruginodis*	*M. brunneum*	*M. brunneum*
						*I. farinosa*	*B. pseudobassiana*
						nematodes	*I. fumosorosea*
							nematodes
Rudegårds alle	55.82	12.45	25	3/3	*M. rubra*	none	*M. brunneum*
							*B. caledonica*
							nematodes
Hestetangsvej	55.81	12.32	31	9/9	*M. rubra*	*M. brunneum*	*M. brunneum*
						*M. flavoviride*	*M. flavoviride*
						*B. bassiana*	*B. pseudobassiana*
						nematodes	nematodes
Logsø	55.84	12.47	46	7/7	*M. ruginodis*	nematodes	nematodes

### 2.2 Molecular identification of entomopathogenic soil fungi

The selected fungal isolates were individually inoculated into sterile flasks containing liquid medium (2% peptone, 3% sucrose and 0.2% yeast extract) and incubated on a shaker (170 r.p.m.) at room temperature for 3 days. The resulting fungal material was filtered under suction and lyophilized overnight. The DNA extraction from dried fungal tissue was carried out using DNeasy blood and tissue kits (Qiagen). Entomopathogenic fungi of the genus *Isaria* were sequenced at the internally transcribed spacer region of the 18S nuclear ribosomal DNA, using the primer pair ITS1 (forward) and ITS4 (reverse) [15,16]. PCRs were carried out in 25 μl volumes. Each reaction contained 1×PCR gold buffer, 4 mM MgCl_2_, 200 μM of each nucleotide, 1 μM of each primer, 1.25 U AmpliTaq Gold^r^ DNA polymerase (Invitrogen), 1 μl template DNA and ddH_2_O added to the total volume. The PCR programme consisted of 5 min initial denaturation at 95°C, followed by 35 cycles of 15 s denaturation at 95°C, 15 s annealing at 55°C and 90 s elongation at 72°C. The reaction ended with a final elongation at 72°C for 7 min and a hold temperature of 4°C.

*Beauveria* and *Metarhizium* spp. were identified by sequencing the 5′ end of the translation elongation factor 1-*α* gene (5′-TEF) using the primers EF2F (forward) and EFjR (reverse) [17]. The PCRs were set up in 50 μl volumes. Each reaction comprised 10 μl Phusion HF buffer (1.5 mM MgCl_2_), 200 μM of each nucleotide, 1 μM of each primer and 0.5 U Phusion high fidelity polymerase (Finzymes), 1 μl template DNA and sterile Milli-Q H_2_O added to the total volume. The PCRs were as follows: 30 s initial denaturation at 98°C, followed by 10 cycles of a touchdown programme with denaturation at 98°C for 10 s and annealing/extension during 90 s. The annealing/extension temperature started at 70°C and was lowered by 1°C per cycle until it had reached 60°C. Thereafter, the reaction continued with 38 cycles of denaturation at 98°C for 10 s, annealing at 60°C for 30 s and elongation at 72°C during 30 s, followed by a final elongation at 72°C for 10 min. All PCR products were quantified in 2% agarose gels, run at 150 V and 100 A for 35 min before sequencing. PCR products were purified using ExoSAP-IT^r^ (Affymetrix) and kit purification GFX DNA/gel band 100 RXN. After purification, the samples were sent to Beijing Genomics Institute or Eurofins MWG for cycle sequencing. The returning chromatograms were edited and assembled in Geneious v. 6.1.6 (Biomatters; http://www.geneious.com/).

In order to identify the entomopathogenic fungi, we compared their 5′-TEF sequences with a set of TEF reference sequences of identified specimens stored at the USDA Agricultural Research Service Collection of Entomopathogenic Fungi (ARSEF). To identify *Beauveria* species, we chose a selection of ARSEF reference sequences (TEF) for the genus *Beauveria* from Rehner *et al.* [18] and a selection of *Beauveria* strains found and described in Meyling *et al.* [17]. Reference sequences for *Metarhizium* sp. were obtained by searching for the single sequence found in BLAST. We then chose three TEF sequences from the ARSEF collection with the highest pairwise identity as references. All sequences were aligned by use of ClustalW alignment [19] in Geneious v. 6.1.6. A phylogram of TEF sequences including the reference sequences was created using Bayesian inference as implemented in MrBayes v. 3.2.2x86 [20,21]. The HKY+G model of sequence evolution [22] was chosen after calculating the likelihood scores for the alignment in the software jModelTest [23] and the corrected Akaike information criterion [24,25]. Two parallel runs of MrBayes were carried out with one cold and three heated chains (chain heats: 1=cold, 0.91, 0.83, 0.77) for five million generations with every 1000 trees sampled. Convergence was assessed by examination of the minimal effective sample size of TL (ESS=1233) and the potential scale reduction factor (=1.000) [26].

### 2.3 Statistical analysis

The statistical comparisons of the total number of pathogen-infected *G. mellonella* larvae per sample in ant nests versus controls and the total number of *G. mellonella* larvae infected with entomopathogenic fungi per sample in ant nests versus control were carried out using generalized linear mixed models (GLMMs) with zero-inflated Poisson errors in the package *glmmADMB* [27] in R v. 3.0.1 [28]. The presence/absence of ants was treated as a fixed effect and ‘sample pair’ (the nest sample and its control) as a random effect to correct for variation in pathogen abundance between sample pairs. An analogous model was repeated to test for the effect of treatment (control, *M. rubra* and *M. ruginodis*) again including ‘sample pair’ as a random effect. Abundance of single pathogen groups in ant nest samples was compared with that in controls using a binomial GLMM (‘logit’ link) in the R package lme4 [29] with 1/0 coding for ant presence/absence. This analysis was carried out at the level of the single *G. mellonella* larvae. ‘Pathogen’ was included as a fixed effect (missing if no pathogen emerged) and ‘sample pair’ as a random effect.

## 3. Results

**Figure 1.**
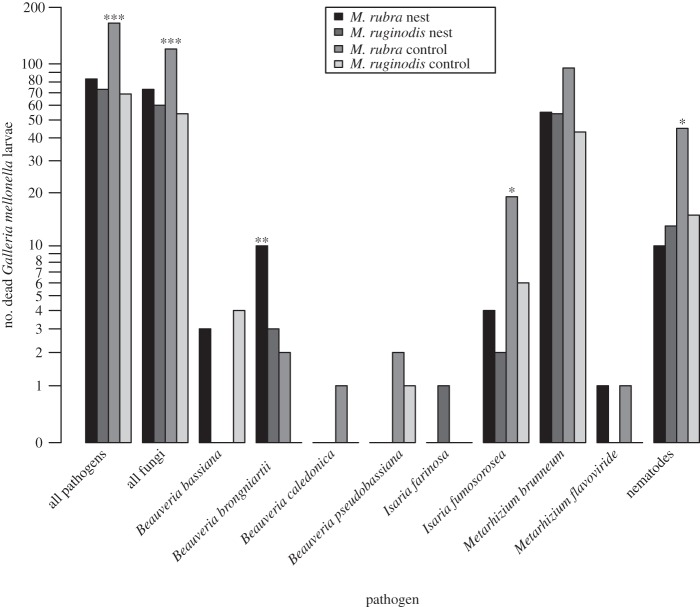
Bar graph shows the total number of *G. mellonella* larvae found per pathogen species or group comparing *M. rubra* and *M. ruginodis* nest samples with their respective controls. Significant differences are marked by asterisks above each set of bars (*p<0.05, **p<0.01, ***p<0.001). The identification of entomopathogenic fungi was carried out by sequencing at either the ITS or 5′-TEF region. For *M. brunneum*, one representative was sequenced per sampling locality.

**Figure 2.**
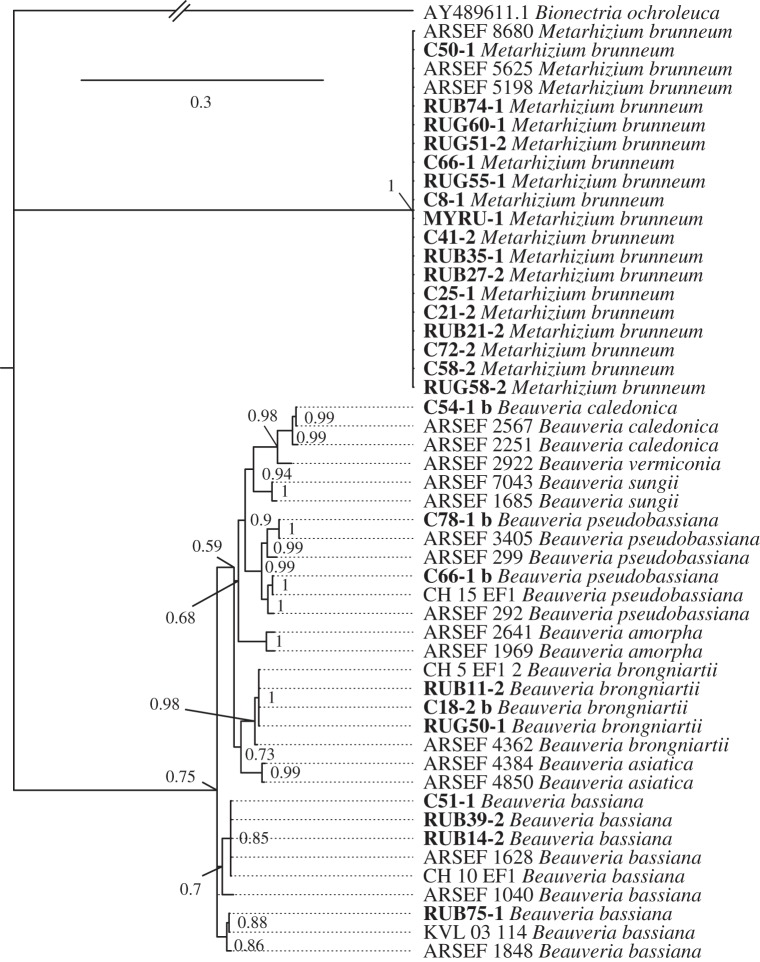
A Bayesian inference phylogram of the 5′-TEF gene of the entomopathogenic fungi sequenced and a set of verified TEF reference sequences from GenBank, representing identified species stored at ARSEF. The phylogram is the consensus tree of 5000 trees obtained from two converged runs in the software MrBayes v. 3.2.2. Node labels show node support (posterior probability ≥0.5). Voucher IDs in bold correspond to samples from this study, those in regular font were used as references obtained from GenBank.

An overview of all isolated entomopathogens, their identification and abundance is given in figures 1 and 2 and tables 1 and 2. The number of *G. mellonella* larvae per sample found dead with a pathogen was lower in soil from ant nests compared with soil from control points (GLMM, nests versus controls: *z*=−4.15, *p*<0.0001). The same was found for entomopathogenic fungi excluding nematodes (*z*=−2.80, *p*=0.005, figure 1). More specifically, *M. rubra* nests contained significantly fewer cadavers with pathogens than the controls (*M. rubra* versus controls: *z*=−4.93, *p*<0.0001, *n*=55 nests), whereas this was not the case for *M. ruginodis* (*M. ruginodis* versus controls: *z*=+0.99, *p*=0.323, *n*=29 nests; figure 1). Several single pathogen species or groups differed significantly in abundance in ant nests compared with controls. Abundance in ant nests was reduced in the fungus *Isaria fumosorosea* (Wize; *z*=−2.51, *p*=0.012) and in entomopathogenic nematodes (*z*=−2.02, *p*=0.044). More surprisingly, the fungus *Beauveria brongniartii* (Saccardo) was more abundant inside than outside ant nests (*z*=+2.89, *p*=0.004), but this was much less abundant than the species that were reduced in ant nests (figure 1), and did not change the overall pattern of decreased *G. mellonella* mortality when exposed to ant nest soil. In all cases, death of a *G. mellonella* larva could be associated with the presence of either a single pathogen or none (i.e. multiple infections were not found).

**Table 2.** Entomopathogenic fungi identified by sequencing: site of collection, treatment (from ant nest or control soil), primers used for amplification, the fungal species, length of the sequenced fragment and GenBank accession number. All sequenced isolates of *Isaria fumosorosea* and *Metarhizium brunneum* had identical sequences.

voucher ID	site	treatment (rug=*M. ruginodis*, rub=*M. rubra*)	primer pair	fungal species	length of sequence (bp)	GenBank accession no.
RUB14-2_b	Allerød Sø	*Myrmica rubra*	EF2F/EFjR	*Beauveria bassiana* (Bals. Criv. Vuill.)	732	KJ908271
C51-1_b	Birkerød	control (rug)	EF2F/EFjR	*Beauveria bassiana*	732	KJ908272
RUB39-2_b	Klampenborgvej	*Myrmica rubra*	EF2F/EFjR	*Beauveria bassiana*	732	KJ908273
RUB75-1_b	Hestetangsvej	*Myrmica rubra*	EF2F/EFjR	*Beauveria bassiana*	732	KJ908274
RUB11-2_b	Allerød Sø	*Myrmica rubra*	EF2F/EFjR	*Beauveria brongniartii*	740	KJ908275
C18-2_b	Allerød Sø	control (rub)	EF2F/EFjR	*Beauveria brongniartii*	740	KJ908277
RUG50-1_b	Birkerød	*Myrmica ruginodis*	EF2F/EFjR	*Beauveria brongniartii*	740	KJ908276
C54-1_b	Rudegards alle	control (rub)	EF2F/EFjR	*Beauveria caledonica* [30]	753	KJ908270
C66-1_b	Allerød forest	control (rug)	EF2F/EFjR	*Beauveria pseudobassiana*[18]	734	KJ908279
C78-1_b	Hestetangsvej	control (rub)	EF2F/EFjR	*Beauveria pseudobassiana*	736	KJ908278
RUG62_i	Allerød forest	*Myrmica ruginodis*	ITS1&4	*Isaria farinosa*(Holmsk.)	495	KJ908284
C12_i	Allerød Sø	control (rub)	ITS1&4	*Isaria fumosorosea*	488	KJ908283
C16_i	Allerød Sø	control (rub)	ITS1&4	*Isaria fumosorosea*	488	KJ908283
C19_i	Allerød Sø	control (rub)	ITS1&4	*Isaria fumosorosea*	488	KJ908283
C20_i	Allerød Sø	control (rub)	ITS1&4	*Isaria fumosorosea*	488	KJ908283
RUB42_i	Dyrehavn	*Myrmica rubra*	ITS1&4	*Isaria fumosorosea*	488	KJ908283
C38_i	Klampenborgvej	control (rub)	ITS1&4	*Isaria fumosorosea*	488	KJ908283
C39_i	Klampenborgvej	control (rub)	ITS5&4	*Isaria fumosorosea*	488	KJ908283
C66-1_m	Allerød forest	control (rug)	EF2F/EFjR	*Metarhizium brunneum*	682	KJ908282
C21-2_m	Allerød Sø	control (rub)	EF2F/EFjR	*Metarhizium brunneum*	682	KJ908282
RUB21-2_m	Allerød Sø	*Myrmica rubra*	EF2F/EFjR	*Metarhizium brunneum*	682	KJ908282
RUG51-2_m	Birkerød	*Myrmica ruginodis*	EF2F/EFjR	*Metarhizium brunneum*	682	KJ908282
C50-1_m	Birkerød	control (rug)	EF2F/EFjR	*Metarhizium brunneum*	682	KJ908282
RUB35-1_m	Gadevang	*Myrmica rubra*	EF2F/EFjR	*Metarhizium brunneum*	682	KJ908282
C72-2_m	Hestetangsvej	control (rub)	EF2F/EFjR	*Metarhizium brunneum*	682	KJ908282
RUB74-1_m	Hestetangsvej	*Myrmica rubra*	EF2F/EFjR	*Metarhizium brunneum*	682	KJ908282
C41-2_m	Klampenborgvej	control (rub)	EF2F/EFjR	*Metarhizium brunneum*	682	KJ908282
C25-1_m	Nymøllevej	control (rub)	EF2F/EFjR	*Metarhizium brunneum*	682	KJ908282
RUB27-2_m	Nymøllevej	*Myrmica rubra*	EF2F/EFjR	*Metarhizium brunneum*	682	KJ908282
RUG55-1_m	Brødeskov	*Myrmica ruginodis*	EF2F/EFjR	*Metarhizium brunneum*	682	KJ908282
RUG58-2_m	Brødeskov	*Myrmica ruginodis*	EF2F/EFjR	*Metarhizium brunneum*	682	KJ908282
C58-2_m	Brødeskov	control (rug)	EF2F/EFjR	*Metarhizium brunneum*	682	KJ908282
RUG60-1_m	Brødeskov	*Myrmica ruginodis*	EF2F/EFjR	*Metarhizium brunneum*	682	KJ908282
C8-1_m	Vaserne	control (rub)	EF2F/EFjR	*Metarhizium brunneum*	682	KJ908282
MYRU-1_m	Vaserne	*Myrmica rubra*	EF2F/EFjR	*Metarhizium brunneum*	682	KJ908282

## 4. Discussion

We found evidence for reduced entomopathogen abundance in nests of *M. rubra* group ants. Especially, *M. rubra* (*n*=55 nests) seems either to reduce the abundance of pathogens inside their nest, or is able to avoid infected nest sites. We cannot distinguish between the two here. Previous studies have shown that some ants do not avoid pathogen-rich nesting sites [31,32], and hygienic behaviour, as observed frequently in ant species including *M. rubra*, such as removal of dead nest-mates to midden piles outside the nest [33], is likely to reduce pathogen abundance. Allogrooming and antimicrobial secretions of the metapleural glands are further thought to prevent pathogens from becoming abundant inside ant nests [34]. It has previously been shown that non-sterile soils from nests of the red imported fire ant *Solenopsis invicta* mitigated effects of *B. bassiana* compared with sterilized soil, suggesting an antagonistic effect of micro-organisms present in the soils of ant nests [35,36]. Similarly, increased microorganismal activity in *Myrmica* ant nests could eventually explain the reduced abundance of entomopathogenic fungi compared with control soil. In nests of *M. ruginodis* (*n*=28), no evidence for a reduction of entomopathogens was found: the number of *G. mellonella* larvae with pathogens did not differ significantly between soil samples from nests and controls, with slightly more entomopathogens inside nests. This may suggest extraordinary tolerance of *M. ruginodis* to entomopathogens, leading to a competitive advantage over other ant species (e.g. *M. rubra*) for *M. ruginodis* in pathogen-rich sites. For myrmecophiles, this could mean that *M. ruginodis* is a less suitable host species than *M. rubra*. Indeed, the myrmecophilous butterfly *Maculinea alcon* has been suggested to use *M. rubra* as a main host and *M. ruginodis* as a secondary host in Denmark [37].

We conclude that this study supports the idea of an immunological benefit of myrmecophily and suggest to test this theory further using facultatively and obligatorily myrmecophilous species.

## Supplementary Material

Data for “Reduced entomopathogen abundance in Myrmica ant nests - testing immunological benefits of myrmecophily using Galleria mellonella as a model” An Excel file containing all data underlying the study.

## Data accessibility

The data used in this study are accessible as electronic supplementary material. DNA sequences are accessible through GenBank.
